# Preparation and Performance Study of Silicone-Oligomer Composite-Modified Polyurethane Sealant

**DOI:** 10.3390/polym17222990

**Published:** 2025-11-11

**Authors:** Ning Li, Feiyu Chen, Qing Liu, Ming Zhao, Cheng Zhang, Peizhe Li, Xueting Ma, Jiangye Zheng, Qunchao Zhang

**Affiliations:** 1 State Grid Beijing Electric Power Cable Company, Beijing 100022, China; 2 School of Materials Science and Engineering, Hubei University, Wuhan 430062, China; 3 Wuhan Gongke Zhitong Engineering Technology Co., Ltd., Wuhan 430223, China

**Keywords:** polyurethane sealant, silicone oligomer (silane-based), moisture-cured, mechanical properties, hydrophobicity

## Abstract

To address the shortcomings of traditional polyurethane (PU) sealants, including inadequate weather resistance, low curing efficiency, and limited environmental performance, this study synthesized a functional silicone oligomer (DQPSi) featuring both dynamic crosslinking and hydrophobic properties via the sol–gel method, which was subsequently incorporated into the polyurethane matrix. The effects of DQPSi content (0–20 wt%) on the properties of silane-modified polyurethane (SPU) sealants were systematically investigated. Results demonstrate that DQPSi significantly enhances the comprehensive performance of the material. At 15% loading, the sealant achieves optimal performance balance: surface-drying time shortens to 110 min (45% reduction), curing rate increases to 1.7 mm/d (112.5% improvement), tensile modulus rises by 14% to 0.88 MPa, elongation at break substantially increases to 420%, and contact angle improves to 78° with markedly enhanced hydrophobicity. Microscopic analyses (SEM, nanoindentation) confirm that these improvements stem from DQPSi forming a uniform interpenetrating network (IPN) structure with the PU matrix, where dynamic Si-O-Si bonds provide rigidity and stress dissipation while hydrophobic groups (methylpropyl) migrate to the surface to form a barrier. However, excessive addition (20%) induces silicone phase separation and over-crosslinking, causing mechanical degradation (tensile strength decreases to 0.70 MPa, elongation at break drops to 331%) and microcrack formation. This research elucidates DQPSi’s reinforcement mechanism and critical loading threshold, establishing theoretical and technical foundations for developing high-performance eco-friendly silane-modified polyurethane sealants.

## 1. Introduction

Polyurethane sealants are critically important across multiple industrial sectors, including construction, transportation, and electronics, due to their excellent elasticity, strong adhesion, and straightforward application process [1,2]. However, as their range of applications expands and demands for long-term service performance grow increasingly rigorous, the inherent limitations of conventional polyurethane sealants have become more evident [3]. Under prolonged exposure to harsh environmental conditions—such as cyclic heat and humidity [4,5] and ultraviolet (UV) radiation [6]—these materials are prone to degradation, exhibiting phenomena such as yellowing, hardening, and cracking, which can ultimately result in seal failure. These issues are primarily attributed to the insufficient stability of key chemical bonds within their molecular structure [7,8,9].

Additionally, the moisture-dependent curing mechanism of many polyurethane sealants often leads to slow surface drying and delayed deep curing [10], significantly extending construction timelines and reducing project efficiency. Furthermore, certain formulations contain residual active monomers [11] or organic solvent [12], which not only contribute to emissions of volatile organic compounds (VOCs) [13] but also present potential health risks to workers and adverse environmental impact [14]. Such characteristics make it difficult to comply with increasingly stringent ecological and safety regulation [15].

Therefore, the development of a new generation of polyurethane sealants that integrate high performance [16], rapid curing [17], superior weather resistance [18], and environmental compatibility [19] has become a major focus of both academic research and industrial innovation [20].

To address the limitations of conventional polyurethane sealants, silane modification technology [21,22] is widely considered a highly promising solution. This approach introduces hydrolysable alkoxysilane groups into polyurethane molecular chains [23,24], enabling the formation of a three-dimensional crosslinked network dominated by high-bond-energy siloxane (Si–O–Si) bonds under moisture-induced conditions [25]. Such modification not only preserves the intrinsic mechanical advantages of polyurethane but also significantly improves its resistance to UV radiation [26], heat and humidity aging [27], and chemical media [28]. Moreover, since the curing process generally produces only low-toxicity alcohols as by-products, the environmental compatibility of the material is substantially enhanced [29].

However, mainstream silane modification strategies—particularly those using monomeric silane coupling agents via end-capping or blending [30,31]—still face considerable challenges. On one hand, achieving uniform dispersion of silane components within the polyurethane matrix remains difficult, often leading to interfacial defects or phase separation [32]. At higher loading levels, these issues can even result in an unexpected decline in mechanical performance [33]. On the other hand, existing modification methods are often designed with a single functionality in mind [34], making it difficult to simultaneously optimize multi-dimensional performance targets such as curing rate [35], rigidity–toughness balance [36], hydrophobicity [37,38], long-term stability [39], and self-healing capability [40].

A critical aspect is the complex non-monotonic dependence of final material properties on silane loading. The lack of a clear understanding of critical thresholds and associated failure mechanisms significantly hinders the rational design and performance breakthroughs in high-performance silane-modified polyurethane (SPU) sealants.

To overcome the aforementioned challenges, this study designed and synthesized a functional siloxane oligomer—designated DQPSi (Dynamic Quasi-Prepolymer Siloxane)—that integrates both dynamic crosslinking capability and hydrophobic characteristics [41,42] and incorporated it into an SPU sealant system. This strategy effectively mitigates the drawbacks of conventional polyurethane sealants, such as inadequate weather resistance, slow curing, and poor environmental compatibility. In contrast to previously reported modification strategies employing silicone oligomers or single silane coupling agents, the key innovation of this work lies in achieving multifunctional synergistic enhancement: DQPSi not only accelerates the moisture-induced curing process due to its abundant hydrolysable groups, but its flexible siloxane segments and hydrophobic alkyl chains also contribute to the formation of a dynamic crosslinked network within the matrix and spontaneously migrate to the surface to establish a hydrophobic barrier, respectively. These combined effects simultaneously improve the mechanical properties, curing rate, and long-term durability of the sealant.

Notably, the modified system achieves a 14% increase in modulus while maintaining a high elongation at break (420%), realizing an optimal stiffness–toughness balance. This resolves the common trade-off in conventional modifications where high crosslinking density and high elasticity are mutually exclusive. Furthermore, this work identifies the critical siloxane oligomer loading level (15 wt%) and elucidates the corresponding phase separation mechanism, thereby providing important theoretical insights and a practical pathway for the molecular design and controllable fabrication of high-performance environmentally friendly sealants. These findings are of significant value for advancing the application of such sealants in demanding fields such as high-end construction and rail transportation.

## 2. Materials and Methods

### 2.1. Materials

Methylpropyldiethoxysilane (MPDES), Isophorone diisocyanate (IPDI), N-β-aminoethyl-γ-aminopropyl trimethoxysilane (KH-792), Vinyltrimethoxysilane (KH-171): technical grade, Hubei Jianghan New Material Co., Ltd., Jingzhou, China; Mercaptomethyltriethoxysilane: laboratory-synthesized; Tetraethyl orthosilicate (TEOS, 97%): Chengdu Kelong Chemical Co., Ltd., Chengdu, China; Trifluoromethanesulfonic acid (98%): Shanghai Aladdin, Shanghai, China; Polypropylene glycol (PPG, Mn 2000): Energy Chemical, Shanghai, China; Activated calcium carbonate (6250 mesh): Shanghai Chenqi Chemical Technology Co., Ltd., Shanghai, China.

### 2.2. Synthesis of DQPSi Silicone Oligomer

A functional siloxane oligomer (DQPSi) was synthesized via a controlled hydrolysis-condensation reaction. Into a four-necked flask equipped with a mechanical stirrer, thermometer, condenser, and dropping funnel were charged 17.6 g (0.1 mol) of MPDES, 10.8 g (0.05 mol) of TEOS, and 30 g of anhydrous ethanol. The mixture was stirred and heated to 60 °C under ambient atmosphere. A solution containing 5.76 g (0.32 mol) of deionized water and 0.3 g of trifluoromethanesulfonic acid catalyst dissolved in a measured amount of ethanol was introduced dropwise into the reaction mixture over a period of 20 min. The reaction was maintained at 60 °C with continuous stirring for 2 h.

**Figure 1:**
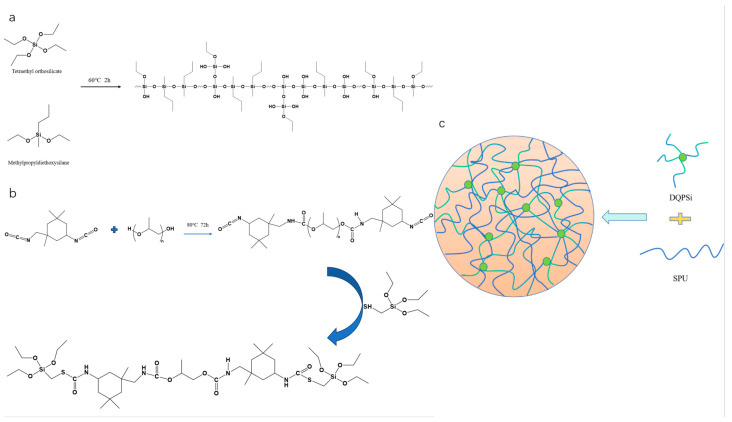
The synthesis of DQPSi and SPU. (**a**) Synthesis of DQPSi Silicone Oligomer. (**b**) Synthesis of Silane-Modified Polyurethane Prepolyme. (**c**) Crosslinked Network Structure of Sealant.

Upon completion of the hydrolysis step, the temperature was elevated to 93 °C to remove ethanol by atmospheric distillation. The system was then subjected to reflux at 90 °C until no further condensate was observed returning from the condenser, indicating termination of the condensation reaction and formation of the target DQPSi oligomer. The synthetic route is schematically presented in Figure 1a.

### 2.3. Synthesis of Silane-Modified Polyurethane Prepolymer

Poly(propylene glycol) (PPG) was first dehydrated in a vacuum oven at 120 °C for 4 h to eliminate residual moisture. A precisely weighed quantity of the dried PPG and isophorone diisocyanate (IPDI) were introduced into a pre-dried four-necked flask. The mixture was heated to 80 °C under a nitrogen atmosphere and maintained under stirring for 72 h to form an isocyanate-terminated polyurethane prepolymer (–NCO–PU). After cooling the reaction system to 60 °C, a measured amount of triethylamine catalyst and mercaptomethyltriethoxysilane were added to conduct an end-capping reaction. The mixture was allowed to react for an additional 4 h. The synthesis process is shown in Figure 1b, after which the residual –NCO content was determined by dibutylamine back-titration. The reaction was considered complete upon the full consumption of –NCO groups, yielding the target silane-modified polyurethane prepolymer (SPU).

Upon exposure to atmospheric moisture, the ethoxysilane groups located at the terminal ends of the prepolymer, as well as any residual ethoxy functionalities present in the siloxane oligomers, undergo hydrolysis followed by condensation, thereby effecting a crosslinked network that enables curing of the sealant. The cross-linked network model is shown in Figure 1c. In the resulting SPU prepolymer structure, PPG constitutes the soft segment, forming the flexible backbone of the polymer chains and conferring elasticity and flexibility to the material. In contrast, IPDI contributes a rigid cyclic structure, which—upon reaction with the end-capping agent (mercaptomethyltriethoxysilane)—forms covalent linkages that, together with the rigid moieties of the end-capping agent itself, constitute the hard segments of the polymer.

### 2.4. Preparation and Optimization of Silane-Modified Polyurethane Sealant

Pre-weighed quantities of active calcium carbonate and PPG-2000 were sequentially charged into a planetary mixer and blended for 0.5 h. Subsequently, a specified amount of vinyl trimethoxysilane(KH-171) was introduced, after which the system was heated to 120 °C and placed under vacuum. Mixing was continued under reduced pressure for 3 h to thoroughly remove moisture. Following cooling to room temperature, the corresponding amounts of N-β-aminoethyl-γ-aminopropyl trimethoxysilane(KH-792) and SPU prepolymer (fixed at 100 phr) were incorporated (Note that here, phr stands for Parts per hundred parts of resin). The mixture was then subjected to vacuum degassing for 0.5 h. Once a homogeneous dispersion of all fillers was achieved, the final product was collected and stored in a sealed container for subsequent use. It is noteworthy that the vacuum environment throughout the process serves to maintain system stability by preventing premature cross-linking, rather than to promote curing.

All specimens were evaluated for tensile strength, elongation at break, and surface drying time in accordance with the standard testing methodology detailed in Section 2.5.

To over; come the limitations associated with conventional single-factor optimization approaches and to quantitatively evaluate the individual and interactive effects of key components on sealant performance, this study employed an orthogonal experimental design—a robust statistical methodology—for systematic formulation optimization. Four critical factors exerting the most pronounced influence on sealant properties were identified: filler (A: active calcium carbonate), plasticizer (B: PPG-2000), desiccant (C: KH-171), and crosslinking agent (D: KH-792). Each factor was assigned three levels, spanning a rationally determined range of concentrations to comprehensively capture their functional effects. The specific factor-level configuration is presented in Table 1.

The experimental plan was arranged using the L9(3^4^) orthogonal table. This table was designed based on the principles of balanced dispersion and orderly comparability. Through only 9 experiments, it can efficiently obtain sufficient information, scientifically analyze the primary and secondary influences of each factor, and optimize the optimal combination. The experimental plan and results are shown in Table 2.

Perform range analysis on the experimental results obtained from Table 2. Calculate the average values (Ki) and ranges (R) of each performance indicator for each factor at different levels. Ki is used to evaluate the optimal level of the factor, while the range R is used to determine the significance of the factor’s impact on the performance indicators.

For each performance index Y (tensile strength, elongation at break, or drying time), let the average response values of factors A, B, C, and D at the jth level (j = 1, 2, 3) be Kij. Among them, the formula for calculating the average response value of the factor levels is as follows:

$$K_{i}j=(1/n_{j})\times \Sigma Y_{i}jk$$

Among them, *i* represents the factor number, *j* represents the level number, *k* represents the experimental group number, and *n_j_* represents the number of experiments conducted for this factor at the jth level.

The formula for calculating the range is as follows:

$$R_{i}=max(K_{i}1,K_{i}2,K_{i}3)-min(K_{i}1,K_{i}2,K_{i}3)$$

Here, *R_i_* denotes the extent of influence exerted by factor *i* on the performance indicator.

The analysis results are shown in Table 3, Table 4 and Table 5, respectively.

The results of the range (R) analysis reveal considerable variation in the influence of different factors on each performance metric. With respect to tensile strength (Table 3), the factors are ranked in the following order of significance: A > C > D > B. The content of active calcium carbonate (A) is identified as the most critical factor governing tensile strength, with the optimal level determined as A2 (90 phr). Regarding elongation at break (Table 4), the order of influence is A ≈ B > C > D, indicating that both filler (A: calcium carbonate) and plasticizer (B: PPG-2000) play comparably dominant roles in determining material toughness. The corresponding optimal levels are A2 (90 phr) and B2 (50 phr), respectively. Notably, the optimal content of desiccant KH-171 (C) is identified as C3 (4 phr).

In the case of surface drying time (Table 5), the significance ranking is B > A ≈ C > D, highlighting the plasticizer PPG (B) as the most influential factor in regulating curing kinetics. The optimal level is B3 (60 phr), suggesting that increasing PPG content effectively accelerates surface drying. Factors A and C exhibit identical R values, indicating a statistically equivalent degree of influence, and are jointly ranked as the second most significant factors.

A comprehensive analysis reveals that the optimal formulations for different performance metrics do not fully coincide, reflecting inherent performance trade-offs within the sealant system. For instance, while the C3 level (KH-171 at 4 phr) contributes to maximizing elongation at break, an excessive amount of this component inhibits the curing reaction and adversely affects surface drying time. Given the core objective of this study—to develop a highly elastic sealant with well-balanced overall properties—elongation at break was selected as the primary performance criterion, while maintaining appropriate curing efficiency and tensile strength.

Consequently, the second experimental formulation (A2B2C2D2) was identified as the optimal compromise, corresponding to: active calcium carbonate 90 phr, PPG-2000 50 phr, KH-171 3 phr, and KH-792 3 phr. This formulation achieved the most favorable balance among the key performance indicators, with an elongation at break of 355%, a tensile strength of 0.81 MPa, and a surface drying time of 205 min, demonstrating overall characteristics superior to those of other tested combinations.

The active calcium carbonate filler and the plasticizer PPG-2000 were introduced into a planetary mixer and mixed uniformly under stirring for 0.5 h. Subsequently, the dehydrating agent KH-171 was added, and the temperature was increased to 120 °C. The mixture was then subjected to vacuum conditions and further mixed for 3 h. After cooling to room temperature, the crosslinking agent KH-792, silane-modified polyurethane resin, and silane oligomer (DQPSi) were incorporated sequentially and stirred for an additional 0.5 h to ensure homogeneity. A silane-modified polyurethane elastic sealant was thereby obtained. According to the GB/T 528-2009 [43] standard, the prepared sample is placed in a dumbbell-shaped polytetrafluoroethylene mold with a length of 115 mm, a working part width of 6.0 ± 0.1 mm (parallel part), and a narrow part standard thickness of 2.0 ± 0.2 mm. Five formulations of the sealant were prepared by varying the mass fraction of the DQ silane oligomer (DQPSi) at 0%, 5%, 10%, 15%, and 20%. The preparation formula for SPU sealant is shown in Table 6,

### 2.5. Characterization

The chemical structures of DQPSi, the SPU prepolymer, and relevant raw materials were characterized by Fourier Transform Infrared Spectroscopy (FT-IR) using a Thermo Fisher CLY10-is50 spectrometer, Thermo Fisher Scientific, Waltham, MA, USA, ^1^H Nuclear Magnetic Resonance (^1^H NMR) analysis of DQPSi was performed on a Bruker AVANCE NEO HGY spectrometer, Bruker, Bilerika, MA, USA, with dimethyl sulfoxide (DMSO) as the solvent. Surface drying time and curing rate were measured in accordance with standards GB/T 13477.5-2002 [44] and GB/T 32369-2015 [45], respectively. Tensile properties were evaluated using I-shaped specimens prepared according to GB/T 13477.8-2017 [46] and tested on a TA CMT4104 universal testing machine, Jinan, China, at a crosshead speed of 10 mm/min, from which tensile strength, elongation at break, and tensile modulus were determined. Nanoindentation tests were carried out on a Nano-mechanic iNano system under a maximum load of 0.5 mN to obtain local elastic modulus and load fluctuation. Hydrophobicity was assessed via water contact angle measurements using a ZJ-6900 goniometer, Shenzhen, China, with results averaged over five independent droplets. The microstructural morphology of fractured surfaces of cured sealant samples was examined using a Malvern JSM6510LV, JEOL, Tokyo, Japan, field-emission scanning electron microscope (SEM). Prior to imaging, samples were sputter-coated with a thin gold layer and observed at an acceleration voltage of 5 kV.

## 3. Results

### 3.1. Structural Characterization of DQPSi and SPU Prepolymer

**Figure 2:**
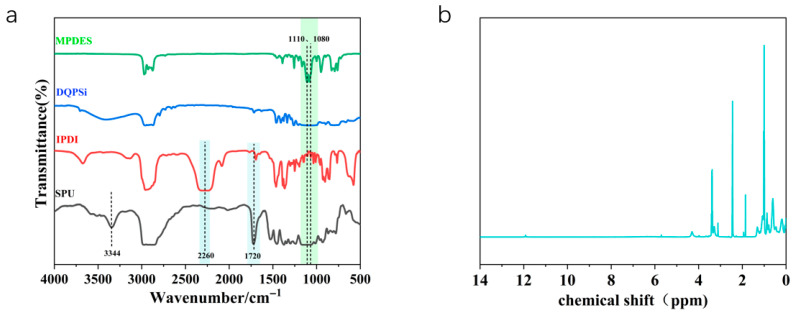
(**a**) FT-IR Spectra of MPDES, DQPSi, IPDI, and SPU; (**b**) ^1^H NMR Spectrum of DQPSi Silane Oligomer.

FT-IR analysis (Figure 2a) confirms the successful synthesis of both DQPSi and the SPU prepolymer. In the spectrum of DQPSi, the characteristic double peak of the Si–OEt group from the precursor MPDES, located at 1110 and 1080 cm^−1^, is replaced by a broad and intense absorption band between 1000 and 1130 cm^−1^, attributable to the asymmetric stretching of Si–O–Si bonds. This shift indicates the completion of hydrolytic condensation and the formation of a siloxane network. In the SPU spectrum, the distinctive –NCO peak of IPDI at 2260 cm^−1^ is no longer detectable. Concurrently, new, strong absorption bands appear at 3344 cm^−1^ and 1720 cm^−1^, corresponding to N–H stretching vibrations and C=O stretching vibrations of the urethane carbonyl group, respectively. These spectral changes provide clear evidence for the consumption of isocyanate groups and the successful formation of urethane linkages (–NH–COO–).

The ^1^H NMR spectrum of DQPSi (Figure 2b) provides further structural confirmation. Resonances observed at 3.2–3.4 ppm are attributed to the methylene protons of Si–O–CH_2_– groups, indicating the presence of residual ethoxy functionalities (–OEt). The signal at 2.5 ppm originates from the solvent DMSO-d_6_. A resonance at 1.9 ppm is assigned to silanol groups (Si–OH), while the multiplet in the 0.5–1.1 ppm region corresponds to methyl (–CH_3_) and methylene (–CH_2_–) protons in the alkyl backbone. The collective spectral features confirm the presence of both hydrolytically active alkoxysilane groups and silanol species, indicating a partially condensed structure. These retained reactive sites (Si–OR and Si–OH) are essential for subsequent moisture-induced hydrolysis and condensation, enabling the construction of a dynamic crosslinked network within the sealant matrix.

In this study, KH-171 was employed as a desiccant and auxiliary crosslinking agent to prevent pre-crosslinking and gelation during storage, as well as to avoid CO_2_ generation from the reaction between water and –NCO groups. The methoxy groups (–OCH_3_) of KH-171 exhibit higher reactivity than the silane groups in SPU, enabling them to preferentially react with and hydrolyze moisture, producing silanol and methanol. This effectively “scavenges” water, protecting the SPU backbone from adverse reactions. Additionally, KH-171 contributes to the construction of a robust siloxane crosslinked network. The silanol (Si–OH) generated by hydrolysis of KH-171 can not only self-condense to form Si-O-Si bonds but also undergo co-condensation with the silanol groups hydrolyzed from the SPU prepolymer, integrating into the entire crosslinked network and enhancing the material’s performance.

The SPU prepolymer serves as the network backbone. The silane groups at its terminals are key to forming the primary crosslinked network. Multiple SPU molecules interconnect via terminal silanol groups (–Si–OH), creating an extensive network structure that provides the primary mechanical strength.

N-β-aminoethyl-γ-aminopropyl trimethoxysilane (KH-792) acts as the main crosslinking agent. Its amino group can react with any residual isocyanate groups (–NCO) in the system, serving a chain extension function. More importantly, its own triethoxysilane moiety actively participates in hydrolysis and condensation reactions, acting as an additional crosslinking point that significantly increases crosslinking density, thereby enhancing modulus and hardness.

Polypropylene glycol (PPG-2000) acts as a plasticizer. Its long flexible chains weaken the interactions between hard segments, enhance chain mobility, thereby increasing elongation at break and making the material more flexible.

Activated calcium carbonate (CaCO_3_) serves as a filler. Its “activated” surface exhibits better compatibility and bonding strength with the organic polymer matrix (SPU), preventing the formation of weak points.

### 3.2. Effect of DQPSi on the Curing Behavior of Sealant

**Figure 3:**
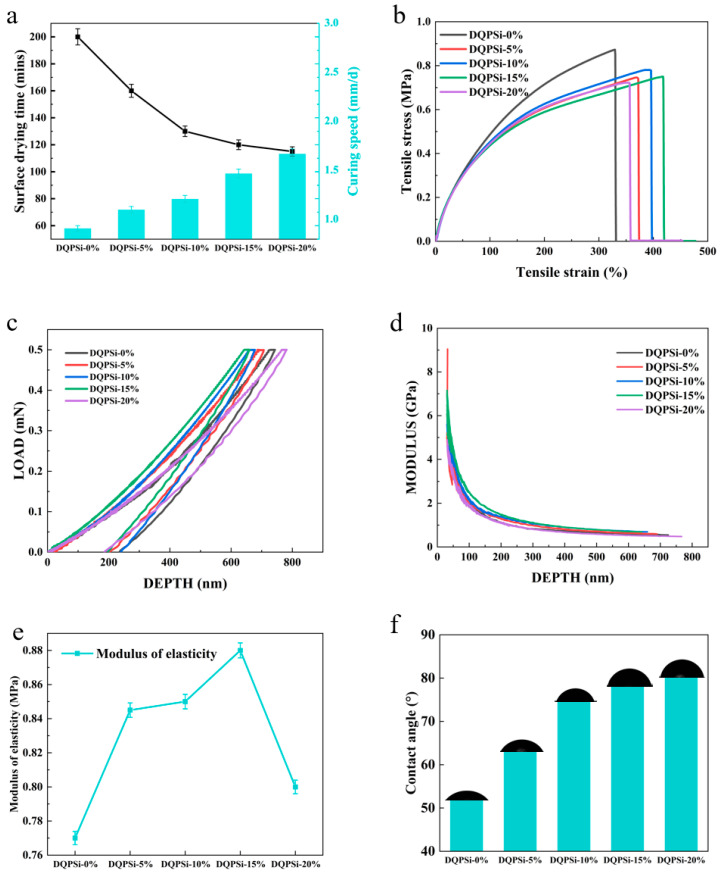
(**a**) Surface Drying Time and Curing Rate of Sealants with Different DQPSi Contents; (**b**) Tensile Strength and Elongation at Break of Sealants with Different DQPSi Contents; (**c**) Maximum Load in Nanoindentation and (**d**) Elastic Modulus at Varied DQPSi Contents; (**e**) Tensile modulus of sealants as a function of DQPSi content; (**f**): Water contact angle of SPU sealants as a function of DQPSi content.

The incorporation of DQPSi markedly accelerates the moisture-induced curing kinetics of the SPU sealant, as illustrated in Figure 3a. Add five groups of DQPSi with different concentrations to the SPU sealant, and mark them, respectively, as DQPSi-0%, DQPSi-5%, DQPSi-10%, DQPSi-15% and DQPSi-20%. With increasing DQPSi content from 0% to 20%, the surface drying time decreased significantly from 200 min to 110 min—a reduction of 45%—while the curing rate increased from 0.8 mm/day to 1.7 mm/day, representing a 112.5% enhancement. This pronounced acceleration is attributed to the presence of hydrolytically active ethoxy (–OEt) and silanol (Si–OH) groups in DQPSi, which serve as additional moisture-reactive sites. Under ambient humidity, these groups undergo rapid hydrolysis to form silanols, followed by condensation into a Si–O–Si crosslinked network. Higher DQPSi loadings increase the concentration of these reactive species, thereby promoting both surface condensation—leading to shortened surface drying time—and the formation of an internal crosslinked structure, which enhances the overall curing rate.

Notably, the rate of curing improvement begins to plateau at DQPSi contents above 15%, with only a marginal increase of 0.2 mm/day observed between the DQPSi-15% and DQPSi-20% formulations. This leveling-off effect can be ascribed to several factors: (1) reduced compatibility at higher loadings, resulting in partial agglomeration and incomplete utilization of reactive sites; (2) excessive surface crosslinking, which forms a dense barrier that hinders further inward moisture diffusion; and (3) the inherent hydrophobicity of the methylpropyl segments in DQPSi, which increasingly retards water permeation at elevated concentrations. These findings collectively indicate that a DQPSi loading between 10% and 15% represents the optimal range for achieving rapid curing without introducing processing-related drawbacks.

### 3.3. Effect of DQPSi on Mechanical Properties of Sealant

The incorporation of DQPSi exerts a distinct nonlinear influence on the mechanical properties of the SPU sealant, as depicted in Figure 3a,b. At a 5% loading level, a slight reduction in tensile strength is observed (0.72 MPa compared to 0.88 MPa for unmodified DQPSi-0%), which may be attributed to insufficient compatibility between DQPSi and the polyurethane hard segments during initial blending, resulting in localized stress concentration due to uneven dispersion.

When the DQPSi content is increased to 10%, a moderate recovery in tensile strength (0.75 MPa) occurs, suggesting the onset of siloxane network formation and the development of partial interpenetration and synergy with PU segments. At 15% loading, while the tensile strength measures 0.72 MPa—marginally lower than that of the unmodified system—the elongation at break reaches a maximum value of 420%. Notably, the tensile modulus, which reflects material stiffness, increases consistently with DQPSi content up to 15%, attaining a maximum value of 0.88 MPa at this loading level—a 14% enhancement over DQPSi-0%.

This mechanical behavior indicates that at 15% loading, the dynamic siloxane network (Si–O–Si) derived from DQPSi forms an effective interpenetrating network (IPN) with the PU matrix, particularly interacting with the soft PPG segments. The flexible Si–O–Si chains work synergistically with the PU soft segments to facilitate molecular chain mobility, thereby significantly improving ductility, as reflected by the high elongation at break. Concurrently, DQPSi serves as additional crosslinking points and contributes inherent structural rigidity, leading to enhanced overall stiffness without compromising toughness. This results in an optimal “stiffness–toughness balance,” which is seldom achieved in conventional modifications.

Nanoindentation results (Figure 3c,d) provide further support: DQPSi-15% exhibits the highest localized elastic modulus and the maximum tolerance to indentation load, confirming the reinforcement effect imparted by the well-dispersed siloxane network.

However, a distinct inflection point accompanied by mechanical deterioration was observed when the DQPSi loading was increased to 20%: tensile strength decreased to 0.70 MPa, elongation at break dropped markedly to 331%, and tensile modulus also declined to 0.80 MPa. This reversal in properties suggests that excessive DQPSi induced substantial phase separation and/or over-crosslinking within the matrix.

**Figure 4:**
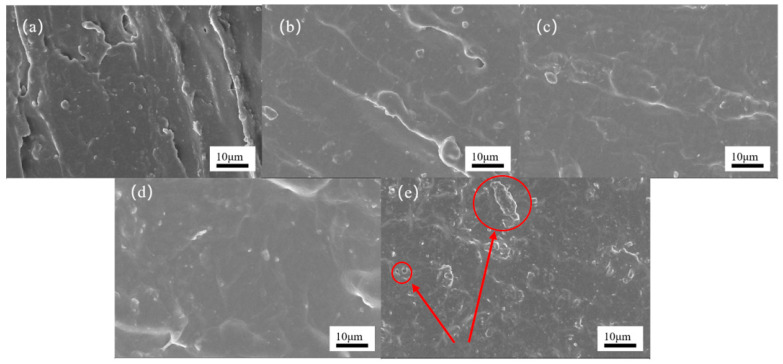
SEM images of SPU sealants with varying DQPSi content: (**a**) DQPSi-0%, (**b**) DQPSi-5%, (**c**) DQPSi-10%, (**d**) DQPSi-15%, (**e**) DQPSi-20%.

Microstructural analysis via SEM (Figure 4) corroborates this interpretation, revealing numerous micron-scale DQPSi aggregates and associated microcracks in the DQPSi-20% sample. These structural defects compromise material continuity, weaken interfacial adhesion, and impede effective stress transfer, ultimately leading to the observed macroscopic degradation in strength, stiffness, and ductility.

Consistent with these findings, nanoindentation results (Figure 3d,e) show that DQPSi-20% exhibits the lowest localized elastic modulus and maximum load tolerance among the modified systems, further evidencing the mechanical degradation induced by microstructural heterogeneity.

This behavior can be rationalized within the framework of percolation theory: when the silane content surpasses a critical threshold (approximately 15% in this system), the material transitions from a homogeneous interpenetrating network to a heterogeneous, phase-separated morphology, resulting in the deterioration of integrated mechanical performance.

### 3.4. Effect of DQPSi on Hydrophobicity of Sealant

The incorporation of DQPSi significantly enhances the surface hydrophobicity of SPU sealants, as evidenced by the progressive increase in water contact angle from 52° (DQPSi-0%) to 79° (DQPSi-20%) with increasing DQPSi loading (Figure 3f). This enhancement is governed by two primary mechanisms: surface segregation of hydrophobic components and formation of a siloxane-rich surface layer.

The hydrophobic alkyl chains derived from Methylpropyldiethoxysilane (MPDES) exhibit strong surface activity due to their low surface energy. During the curing process, these segments spontaneously migrate toward the polymer-air interface to minimize the system’s surface energy. Concurrently, the inherent incompatibility between the hydrophobic DQPSi segments and the polar polyurethane matrix drives microphase separation, further promoting the surface enrichment of hydrophobic groups. This surface accumulation effectively shields the underlying hydrophilic polyurethane segments, resulting in significantly improved hydrophobicity.

At lower DQPSi loadings (5–10%), the surface migration of hydrophobic groups dominates the contact angle enhancement, leading to rapid improvement in hydrophobicity. As the loading increases to 15–20%, the formation of a dense siloxane crosslinked network at the surface becomes the predominant factor, while the contribution from additional hydrophobic group migration reaches saturation, resulting in a more gradual increase in contact angle.

Notably, although DQPSi-20% achieves the highest contact angle (79°), the presence of internal phase separation-induced defects observed in Figure 3e may compromise the long-term stability of the hydrophobic layer and interfacial adhesion. In contrast, SPU-Si-15 maintains excellent mechanical properties while achieving a high contact angle of 78°, representing a more balanced combination of hydrophobicity and structural integrity.

The unmodified SPU sealant (DQPSi-0%) exhibits a water contact angle of 52°. This indicates a strongly hydrophilic surface. Water can easily wet and potentially penetrate the material, resulting in poor waterproofing and weather resistance. After incorporating DQPSi, the contact angle increases to 78°. Although 78° is still classified as “hydrophilic” in absolute terms (<90°), it represents a massive and significant enhancement in hydrophobicity compared to the original 52°.

Despite being below 90°, this improvement brings a qualitative leap in functional performance. It achieves a transition from “non-waterproof” to “effectively waterproof.” The hydrophobic alkyl chains (e.g., methyl, propyl) on the DQPSi molecules spontaneously migrate to the material surface during the curing process.

These groups cover the originally hydrophilic polyurethane segments, reducing the surface energy and thereby increasing the contact angle.

This change in surface chemistry, driven by the migration of surface-active components, is an example of a “hydrophobic modification” mechanism.

## 4. Conclusions

This study successfully developed a functional siloxane oligomer (DQPSi) through a sol–gel process and incorporated it into a silane-modified polyurethane (SPU) sealant system. The optimized base formulation consisted of SPU (100 phr), active calcium carbonate (90 phr), PPG-2000 (50 phr), KH-171 (3 phr), and KH-792 (3 phr). Systematic investigation revealed that DQPSi content exerts a significant influence on sealant properties, with a critical threshold observed at approximately 15% loading.

At this optimal concentration, DQPSi forms a homogeneous interpenetrating network within the PU matrix, leading to synergistic enhancement of comprehensive performance: surface drying time was markedly reduced to 110 min (representing a 45% reduction), while the curing rate increased to 1.7 mm/d (a 112.5% improvement). The material demonstrated a 14% increase in tensile modulus (0.88 MPa) while achieving an exceptional elongation at break of 420%, representing an optimal stiffness-toughness balance. Additionally, the water contact angle increased to 78°, indicating significantly enhanced surface hydrophobicity.

However, excessive DQPSi incorporation (20%) induced siloxane phase separation, as confirmed by SEM observations of aggregated structures, along with over-crosslinking phenomena. These microstructural defects resulted in comprehensive deterioration of mechanical properties (strength, modulus, and elongation) and promoted microcrack formation.

The present work elucidates the enhancement mechanisms and critical threshold effects of siloxane oligomers in polyurethane sealants, providing important insights for the development of high-performance, environmentally friendly sealing materials.

## Figures and Tables

**Table 1:** Orthogonal Experimental Factors and Levels.

Level	A: Active Calcium Carbonate (phr)	B:PPG-2000 (phr)	C: KH-171 (phr)	D: KH-792 (phr)
1	80	40	2	2
2	90	50	3	3
3	100	60	4	4

**Table 2:** L9(3^4^) Orthogonal Experimental Design and Results.

Level	A: Active Calcium Carbonate (Phrase)	B:PPG-2000 (phr)	C: KH-171 (phr)	D: KH-792 (phr)	Tensile Strength (MPa)	Strain at Break (%)	Drying Time (Minutes)
					First/Second/Third/Standard Deviation (MPa)	First/Second/Third/Standard Deviation (%)	First/Second/Third/Standard Deviation (min)
1	80	40	2	2	0.78/0.76/0.79/0.015	325/319/326/3.79	235/241/231/5.03
2	80	50	3	3	0.81/0.80/0.79/0.012	355/349/357/4.16	205/211/199/6.00
3	80	60	4	4	0.75/0.77/0.76/0.013	370/359/374/7.77	185/201/189/8.33
4	90	40	3	4	0.86/0.84/0.87/0.015	345/337/353/8.0	220/233/221/7.23
5	90	50	4	2	0.83/0.81/0.84/0.017	395/393/386/4.73	195/207/193/7.57
6	90	60	2	3	0.79/0.77/0.80/0.015	360/371/364/5.57	210/212/215/2.52
7	100	40	4	3	0.73/0/71/0.74/0.012	310/317/305/6.03	245/237/239/4.16
8	100	50	2	4	0.76/0.75/0.77/0.012	335/329/331/3.06	230/217/224/6.51
9	100	60	3	2	0.80/0.78/0.81/0/015	350/339/361/11.0	200/211/206/5.51

**Table 3:** Range Analysis Table for Tensile Strength.

Index	Factor	K1	K2	K3	R (Range)	Order of Priority
Tensile strength (MPa)	A: Calcium carbonate	0.780	0.827	0.763	0.064	1
	B: PPG	0.790	0.800	0.780	0.020	4
	C:KH-171	0.777	0.823	0.770	0.053	2
	D:KH-792	0.803	0.777	0.790	0.026	3

**Table 4:** Analysis Table of the Range of Elongation at Break.

Index	Factor	K1	K2	K3	R (Range)	Order of Priority
Elongation at break (%)	A: Calcium carbonate	350.0	366.7	331.7	35.0	1
	B: PPG	326.7	361.7	360.0	35.0	2
	C:KH-171	340.0	350.0	358.3	18.3	3
	D:KH-792	356.7	341.7	350.0	15.0	4

**Table 5:** Analysis Table of the Range of Variation in Drying Time of the Sample.

Index	Factor	K1	K2	K3	R (Range)	Order of Priority
Drying time (min)	A: Calcium carbonate	208.3	208.3	225.0	16.7	2
	B: PPG	233.3	210.0	198.3	35.0	1
	C:KH-171	225.0	208.3	208.3	16.7	3
	D:KH-792	210.0	220.0	211.7	10.0	4

**Table 6:** SPU Sealant Formula.

Factor	DQPSi-0% (phr)	DQPSi-5% (phr)	DQPSi-10% (phr)	DQPSi-15% (phr)	DQPSi-20% (phr)
Calcium carbonate (phr)	90	90	90	90	90
PPG-2000 (phr)	50	50	50	50	50
SPU (phr)	100	100	100	100	100
KH-171 (phr)	3	3	3	3	3
KH-192 (phr)	3	3	3	3	3
DQPSi (phr)	0	13	27	43	62

## Data Availability

The original contributions presented in this study are included in the article. Further inquiries can be directed to the corresponding author.
